# Spinal Epidural Abscess Complicated by Meningitis, Sepsis and Thrombocytopenia in a Patient Lacking Traditional Risk Factors

**DOI:** 10.5811/cpcem.2016.12.33001

**Published:** 2017-03-15

**Authors:** Christian Spano, Michael Ward, Nicole Zagelbaum

**Affiliations:** * Orange Regional Medical Center, Department of Emergency Medicine, Middletown, New York; † Touro College of Osteopathic Medicine, Middletown, New York

## Abstract

Spinal epidural abscess is a rare diagnosis with a classic triad of fever, spinal pain and neurologic deficits. Only a small proportion of patients have all three findings, making the diagnosis challenging. Here we present a case of cervical and thoracic spinal epidural abscess complicated by meningitis, sepsis and thrombocytopenia in a patient lacking traditional risk factors. The patient was initially treated non-operatively secondary to thrombocytopenia but subsequently required transfer to a tertiary care facility for surgical drainage after clinical deterioration. This case report highlights the need for a high index of suspicion and low threshold for imaging when considering this rare but potentially deadly condition.

## INTRODUCTION

Epidural abscesses remains a diagnosis of concern among emergency physicians, as they often present with non-specific symptoms that can quickly progress to permanent disability and death if not identified and treated early.^1^ Pre-disposing risk factors include a history of intravenous (IV) drug use, immunodeficiency, skin and soft tissue infections, trauma, and degenerative joint disease.^2^ Iatrogenic causes have been identified to be a result of procedures including neurostimulation, epidural injections, and catheterization.^3^–^5^ Although the diagnosis remains rare, the number of confirmed cases has doubled over the past two decades from one of every 20,000 admissions to one of every 10,000.^2^ Explanations for the dramatic rise include better detection via magnetic resonance imaging (MRI) and increased use of invasive spinal instrumentation, as well as increasing IV drug use.^6^,^7^

The most common presenting symptoms include back pain and fever, which progress to focal neurologic deficits and bowel/urine incontinence.^2^,^8^ Existing case reports have outlined rare presenting symptoms including abdominal pain, anterior chest pain, and cauda equina syndrome highlighting the frequent delay in diagnosis.^9^–^11^ Here we present a case of a healthy white male lacking traditional risk factors who presented to our community emergency department (ED) with a chief complaint of shortness of breath who was found to have a cervical and thoracic spinal epidural abscess that was complicated by meningitis, sepsis and thrombocytopenia, thwarting immediate surgical decompression.

## CASE REPORT

The patient was a 65-year-old healthy, white, married, non-drinking, non-smoking educated male who presented to our ED complaining of generalized illness and shortness of breath. He reported experiencing generalized fatigue with rapid breathing over the prior 2 – 3 days but denied any cough, lower extremity edema, rashes or chest pain. He reported feeling “feverish” but did not take his temperature during the course of his illness. Although the patient didn’t initially complain of neck or back pain, review of systems revealed mild neck discomfort with bilateral hand paresthesias, but the patient quickly dismissed these symptoms as related to chronic neck pain exacerbated by increased physical activity over the prior one week. He also reported a mild headache. The patient’s son reported that he appeared to be confused at times over the preceding week. The patient denied any previous medical history and his surgical history was only significant for a transmetatarsal amputation one-year prior for a traumatic foot injury. The patient denied any history of IV drug use.

Vital signs of note included a rectal temperature of 100.4, oxygen saturation of 94% on room air, a heart rate of 110 beats per minute and a respiratory rate of 22 respirations per minute. His lungs were clear to auscultation and he did not have any cardiac murmurs. Skin exam revealed a chronic-appearing draining sinus at the site of a previous transmetatarsal amputation of the right foot. There was no surrounding cellulitis or purulent drainage from the wound, which was draining serous fluid at time of exam. His strength and reflexes were normal, but sensation to light touch was decreased along the C5 and C6 dermatome bilaterally. Neck exam revealed nuchal rigidity. There was no midline bony tenderness on the cervical, thoracic or lumbar spine.

**Image 1:**
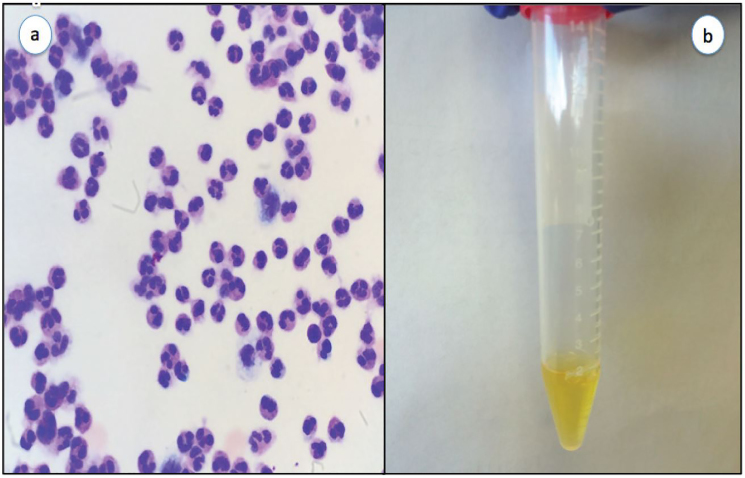
a) Slide of CSF demonstrating many polymorphonuclear leukocytes; b) CSF of patient showing xanthochromia. *CSF,* cerebrospinal fluid

Initial ED laboratory evaluation was significant for leukocytosis of 20.7 10^3^/uL with 80% neutrophils and 12% bands, and thrombocytopenia of 71 10^3^/uL. The patient’s basic metabolic profile was unremarkable with a glucose of 126 mg/dl. Given the patient’s fever, nuchal rigidity and reported confusion by family members, meningitis remained high on the differential diagnosis. A lumbar puncture was performed in the ED, which yielded cloudy cerebral spinal fluid (Image 1b) with a glucose of 10 mg/dL, white blood cell count of 1670 cells/uL and red blood cell count of 78 cells/uL. Cerebrospinal fluid microscopy revealed many polymorphonuclear leukocytes (Image 1a) but the Gram stain was reported to be negative. A computed tomography of the brain was performed prior to lumbar puncture, which was negative. The patient was placed on broad spectrum antibiotics and admitted to the intensive care unit.

**Image 2:**
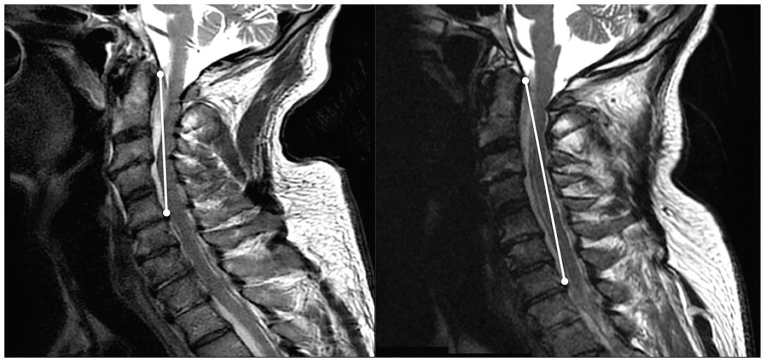
Left: C spine sagittal T2 weighted image taken on day of presentation with lesion from C2–C5. Right: C spine sagittal T2 weighted image 3 days post-admission with larger lesion now from C2–C7.

Although the patient lacked any classic risk factors for epidural abscess, an emergent MRI of the cervical and thoracic spine was ordered from the ED due to the patient’s decreased bilateral upper extremity sensation, which couldn’t be explained by meningitis alone. The MRI revealed an 8 cm epidural abscess involving C2 through C6 (Image 2).

Vancomycin and piperacillin/tazobactam were administered in the ED. An emergent consult was placed to neurosurgery but surgery was deferred secondary to thrombocytopenia. Blood and urine cultures on day two grew methicillin-sensitive *Staphylococcus aureus*. The serous fluid draining from the patient’s chronic foot wound was never Gram stained or cultured.

Over the subsequent 48 hours after admission, the patient’s leukocytosis and thrombocytopenia worsened and a follow-up MRI revealed an extension of the abscess to 12 cm. Clinically, the patient began to have alternating episodes of apnea and tachypnea. As a result of the patient’s deteriorating condition, he was transferred to a tertiary care facility for planned surgical drainage.

## DISCUSSION

Our patient presented to a community ED complaining only of generalized illness and rapid breathing, which was likely the result of the patient’s sepsis with systemic inflammatory response syndrome at the time of presentation. Upon further evaluation, the patient was found to have clinical and laboratory signs of meningitis. The patient’s neurologic complaints elicited on review of systems and mild decreased upper extremity sensation on exam were confounded by his history of chronic neck pain and almost dismissed, but they prompted an MRI in the ED confirming the diagnosis of cervical and thoracic spinal epidural abscess. Our patient was not an IV drug user and was previously healthy and lacking traditional risk factors for this relatively rare diagnosis. He did, however, have a chronically draining wound at a previous transmetatarsal amputation site that we presume was the source of the patient’s bacteremia, causing hematogenous seeding to the epidural space.

## CONCLUSION

Given the rarity of spinal epidural abscesses, medical professionals rely on retrospective case series and case reports as the basis for the scientific literature on this topic. Symptoms are often atypical and the diagnosis of spinal epidural abscess is often delayed. Our case highlights the need for emergency physicians to have a high index of suspicion and a low threshold for imaging to evaluate for spinal epidural abscesses even in patients lacking traditional risk factors and without classic presentations, as a missed or delayed diagnosis can have devastating consequences.
